# Associations Between Recent Contraceptive Use and First Sex Behaviors of Scottish Adolescents: A Brief Report

**DOI:** 10.1080/19317611.2024.2327360

**Published:** 2024-03-31

**Authors:** Malachi Willis, Judith Mabelis, Dorothy Currie, Judith Brown, Joanna Inchley

**Affiliations:** a MRC/CSO Social and Public Health Sciences Unit, School of Health and Wellbeing, University of Glasgow, Glasgow, UK; b School of Medicine, University of St Andrews, St Andrews, UK

**Keywords:** sexual health, contraceptive use, school survey, HBSC

## Abstract

**Objectives:**

We examined associations between recent contraceptive use and first-sex behaviors (early initiation, substance use, contraceptive use) among adolescents in Scotland.

**Methods:**

We used data from the Health Behavior in School-aged Children study.

**Results:**

Controlling for early initiation and substance use, girls and boys who used contraceptives at first sex were 7.5 and 12.3 times more likely to use contraceptives at most recent sexual intercourse than adolescents who did not (*p* < .001). We also present preliminary evidence on contraceptive use of Scottish adolescents in 2022.

**Conclusions:**

Experiences during adolescents’ first sex may have lasting implications for later sexual behavior.

## Introduction

Unintended pregnancy and sexually transmitted infections (STIs) remain relevant among young people in Scotland. Evidencing this, abortion rates increased by 19% from 2021 to 2022 among Scottish adolescents who were 16–19 years old, and cases of gonorrhea and chlamydia infections have risen since 2019 among sexually active people 24 years old or younger (Public Health Scotland, 2022). In the UK, more broadly, 18–24-year-olds in 2020–2021 were more likely than older age groups to have had a pregnancy that was unplanned, to have terminated a pregnancy in the past year, and to have used STI-related services (including tests for chlamydia or HIV) in the past year (Mitchell et al., 2023). For these reasons, the Scottish Government continues to prioritize reducing unintended teenage pregnancy and STI transmission (Public Health Scotland, 2022).

Effective contraceptive use is key to preventing unintended pregnancy and STIs (Beksinska et al., 2020; Steiner et al., 2021), and there is evidence that a precedent for contraceptive use might be set early on: adolescents who use contraceptives during their first sex are more likely to use contraceptives during their most recent sex (Sprecher et al., 2019). For example, a nationally representative longitudinal survey of sexually active adolescents in the United States found that using condoms at first sex was associated with greater odds of condom use at most recent sex—which was 7 years later, on average (Shafii et al., 2004, 2007). Reflecting this increased risk, evidence from adolescents in the UK suggested that not using contraception at first sex is associated with higher likelihood of subsequent pregnancy (Parkes et al., 2009). And in a sample of women aged 18–45 from Denmark, Norway, and Sweden, not using contraception at first sex was significantly associated with having >10 lifetime sexual partners, induced abortions, and STIs (Guleria et al., 2019). In addition to not using effective contraception at first sex, risky sexual behaviors in adolescence include early initiation and substance use at first sex (Symons et al., 2014; Willis et al., 2021).

Indeed, the first time a person experiences sex tends to be a salient event that can be associated with other aspects of a person’s sexual development (Marcantonio et al., 2020; Reissing et al., 2012). Given the lack of research in this area within a Scottish context, we investigated whether risk factors associated with first sex are predictive of adolescents’ recent contraceptive use in a sample of Scottish adolescents—which constitute a subpopulation that continues to be at higher risk of unintended pregnancy than comparable western countries, particularly in the most deprived areas of Scotland (Public Health Scotland, 2022).

H1: Early initiation of sexual intercourse would be negatively associated with contraceptive use at most recent sexual intercourse.

H2: Substance use at first sexual intercourse would be negatively associated with contraceptive use at most recent sexual intercourse.

H3: Controlling for early initiation and substance use at first sexual intercourse, contraceptive use at first sexual intercourse would be positively associated with contraceptive use at most recent sexual intercourse.

## Method

### Participants and Procedure

Data were collected as part of the Health Behavior in School-aged Children (HBSC) study in Scotland. Conducted every 4 years in member countries, the HBSC study is a World Health Organization Collaborative Cross-National Survey. The survey is administered in schools to a nationally representative sample of 11-, 13-, and 15-year-olds and collects data on health and health behaviors, as well as a range of school and social factors. In each survey year, the class is the primary sampling unit, stratified by school grade, and the sample is proportionally stratified by school funding (Local Education Authority [LEA] funded or independent) and by education authority (for LEA funded schools). Ethical approval for each survey round was granted by the university hosting the national HBSC team. See Inchley et al. (2020) for further details regarding methodology.

The present analysis only included 15-year-olds; other age groups did not receive the sexual health items. Specifically, we focused on 15-year-olds who reported having had sexual intercourse in the 2014 and 2018 HBSC Scotland surveys (i.e. 989 out of the 4,099 adolescents who responded to this item). In 2014, 27.3% of girls (*n* = 370) and 24.9% of boys (*n* = 335) reported having had sexual intercourse, and in 2018, 19.7% of girls (*n* = 141) and 21.0% of boys had had sexual intercourse (*n* = 143). Across these two data collections, respondents included in the present study were similar regarding age (mean of 15.7 years in 2014 and 2018) as well as whether their schools were in urban areas (2014: 69.6%; 2018: 64.8%) or were non-denominational regarding religious affiliation (2014: 85.5%; 90.5%). In 2018 only, adolescents who had previously engaged in sexual intercourse were asked about their sexual attraction; 86.8% reported being only attracted to the other gender. For additional exploratory analyses, we also examined data from the 2022 study, in which 19.4% of girls (*n* = 75) and 22.3% of boys (*n* = 85) reported having had sexual intercourse.

### Measures

Our primary outcome measure was whether adolescents had used contraception during their most recent sexual intercourse. Students were separately asked about condoms and birth control pills (0 = neither; 1 = either or both). Risky behaviors measured included early initiation (0 = ≤14 years at first sexual intercourse; 1 = ≥15 years), substance use at first sexual intercourse (0 = no substance use; 1 = alcohol or other drugs), and—in 2018 alone—contraceptive use at first sexual intercourse (0 = no contraceptive use; 1 = condoms, birth control pills, or other contraceptives).

### Analysis

To assess whether risky first sexual intercourse behaviors (i.e. early initiation and substance use) were associated with recent contraceptive use, we conducted chi-squared tests of independence. We reported Cramér’s V (φ_C_) as an effect size for the chi-squared tests. According to Cohen (1988), a φ_C_-value of .1 indicates a small effect size, .3 medium, and .5 large. Next and using 2018 data only, binary logistic models regressed recent contraceptive use onto students’ contraceptive use at first sexual intercourse (controlling for early sexual initiation and substance use at first sexual intercourse); these models were tested separately by gender because girls and boys tend to report discrepant first sexual intercourse experiences (Symons et al., 2014). All tests of significance were conducted at an α-level of .05. Data preparation and analyses were conducted using SPSS 27.

## Results

### Descriptive Statistics

Of those 15-year-olds who reported having had sexual intercourse, we found that 70–75% in 2014 and 2018 used contraceptives at their most recent sexual intercourse (Table 1). Specifically, 55%–60% of Scottish adolescents reported using condoms, and 25%–35% used contraceptive pills. Regarding early initiation, 40%–45% of girls and 45%–54% of boys first had sexual intercourse at 14 years or younger. Rates of using alcohol or drugs at first sexual intercourse were 14%–17% of girls and 20%–24% of boys. In 2018, 74% of girls and 56% of boys used contraceptives at first sexual intercourse.

**Table 1.** Descriptive statistics of adolescent’s contraceptive use at most recent sex and risky first sex behaviors.

	2014 girls (*n* = 370)	2018 girls (*n* = 141)	2014 boys (*n* = 335)	2018 boys (*n* = 143)
*Most recent sex*				
Used condom	59.0%	55.0%	60.8%	60.6%
Used pill	32.0%	35.0%	27.3%	21.5%
Used condom or pill	73.8%	73.2%	73.2%	72.4%
*First sex*				
Age				
≤14	52.0%	44.4%	59.4%	48.4%
≥15	48.0%	55.6%	40.6%	51.6%
Used substances				
Yes	14.4%	17.4%	24.3%	20.0%
No	85.6%	82.6%	75.7%	80.0%
Used contraceptives				
Yes	—	73.5%	—	56.2%
No	—	26.5%	—	43.8%

### Hypothesis Testing

#### Early initiation (H1)

Engaging in first sexual intercourse prior to the age of 15 was negatively associated with recent contraceptive use. In 2014, only 64.5% of boys whose first sexual intercourse occurred when they were 14 or younger used contraception at their most recent sexual intercourse, compared with 83.9% of boys who were at least 15 when they first had sexual intercourse, χ^2^ = 13.50, *p* < .001, φ_C_ = .216. For girls in 2014, this association was non-significant but in the same direction: 69.9% of those with early sexual initiation used contraception at their most recent sexual intercourse compared with 78.4% of girls without early sexual initiation, χ^2^ = 3.32, *p* = .069, φ_C_ = .096. In 2018, these effect sizes were larger for boys (61.4% versus 83.3%, χ^2^ = 7.49, *p* = .006, φ_C_ = .247) as well as girls (67.2% versus 81.1%, χ^2^ = 3.41, *p* = .065, φ_C_ = .159).

#### Substance use (H2)

Using alcohol or other drugs at first sexual intercourse was negatively associated with recent contraceptive use. In 2014, only 48.3% of boys who used substances at first sexual intercourse used contraception at their most recent sexual intercourse, compared with 79.5% of boys who did not use substances, χ^2^ = 22.95, *p* < .001, φ_C_ = .283. This association attenuated for boys in 2018: 60.9% versus 76.0%, respectively, χ^2^ = 2.18, *p* = .140, φ_C_ = .133. Again, this association was non-significant but in the same direction for girls (2014: 66.0% of those who used substances at FTI versus 74.9% of those who did not, χ^2^ = 1.76, *p* = .185, φ_C_ = .071; 2018: 59.1% versus 77.9%, respectively, χ^2^ = 3.45, *p* = .063, φ_C_ = .160).

#### Contraceptive use (H3)

To test the association between contraceptive use at first sexual intercourse and recent contraceptive use, we only included adolescents who reported having had sexual intercourse more than once before (*n* = 220). Controlling for the effects of early sexual initiation and substance use at first sexual intercourse, using contraceptives at first sexual intercourse significantly and positively predicted whether Scottish adolescents in 2018 used contraceptives during their most recent sexual intercourse. Specifically, girls were 7.5 times as likely (*p* < .001) and boys 12.3 times as likely (*p* < .001) to have used contraceptives at most recent sexual intercourse if they had used contraceptives at first sexual intercourse [OR 95% CI for girls: 2.85–19.84; OR 95% CI for boys: 4.23–37.57].

### Exploratory Analysis

Using the 2022 HBSC Scotland survey, we explored rates of contraceptive use during most recent sexual intercourse. We found decrements in the percentage of students reporting condom use in 2022 (41.8% of girls; 53.2% of boys), but boys reported higher rates of using contraceptive pills (34.7%) than they had in previous years compared with a relatively stable 31.9% for girls (see Table 1). The only risky first sexual intercourse behavior assessed in the 2022 HBSC Scotland survey was early initiation. Similar to 2014 and 2018, 52.0% of sexually active girls and 48.8% of sexually active boys in 2022 had engaged in sexual intercourse by the time they were 14.

## Discussion

Our findings suggest that a precedent for risky sex may be set early on in adolescence—corroborating other evidence that first sexual experiences can predict later sexual behavior and wellbeing (e.g. Guleria et al., 2019; Shafii et al., 2007). As such, effective sexual health education is needed as primary prevention to teach young people about safe sex practices before they begin engaging in sexual activity. Adolescents should not be left to learn critical strategies for maintaining their sexual health and wellbeing on their own or from informal sources like friends and mass media. Demonstrating a step forward in the UK, Relationships and Sex Education (RSE) recently became compulsory for students at secondary schools in England from September 2020 (Department of Education, 2021). However, such statutory guidance still does not exist for Scotland and the current guidance related to national standards does not address sexual risk or contraception (Education Scotland, 2022), despite unintended pregnancy and STIs remaining relevant among Scottish adolescents (Public Health Scotland, 2020, 2022).

Further emphasizing the ongoing and increasing need for effective primary prevention via widespread sexual health education, preliminary data from the 2022 HBSC survey provides evidence of less condom use among Scottish adolescents. While it is encouraging that more boys reported that their partner used oral contraception—potentially suggesting greater sexual communication or awareness among young people, the corresponding decrease in condom use is concerning because young people might think that oral contraceptive pills protect them from STIs in addition to preventing pregnancy. Sexual health education must emphasize that condoms are required to effectively prevent STIs. Decrements in condom usage may have also been due to restricted access during pandemic-related lockdown measures; these are some of the first national data on first sex of adolescents during the era of the COVID-19 pandemic. A nationally representative sample of adults in the UK found that many—especially younger people—experienced difficulty accessing condoms during the first few months of the pandemic (Dema et al., 2022). That patterns of sexual intercourse and early sexual behavior among Scottish adolescents persisted in 2022 suggests that opportunities to sexually express themselves continued during extended periods of social distancing—indicating their ongoing need to access safe and reliable contraception.

These data may be limited in how well they represent young people’s sexual experiences and gender identity. For example, asking about sexual intercourse may discount nonheterosexual sexual activity or other sexual behaviors that are relevant to adolescent sexual health. HBSC researchers are determining how to revise items to better capture young people’s sex, gender identity, and sexual orientation (Költő et al., 2020). Another limitation of these data is that of memory biases, which can influence people’s self-reported sexual behavior. But by using a sample of adolescents, these biases might be reduced in HBSC relative to other studies that have relied on asking university students or even older participants to reflect on first time sexual experiences that happened years or even decades previously. A limitation to sampling 15-year-olds is that only a minority reported having had sexual intercourse. As such, we acknowledge that our findings may not be generalizable to older adolescents.

Given that early sexual risk can set a precedent for later sexual risk, early adoption of healthy sexual attitudes and behaviors is critical. To support the sexual health and reproductive autonomy of adolescents, effective contraception must be widely and freely available—as it currently is in the UK even for adolescents under 16 and without needing the consent of a parent or guardian (NHS, 2021). Unrestricted access to the safest and most reliable contraceptive methods (e.g. hormonal pills, long-acting reversible contraception) must be complemented by education that the need to protect oneself sexually does not end with contraception; young people should still be encouraged to use condoms to decrease their risk of contracting an STI. Overall, the lasting effects of early risky sexual behavior leading to later risk demonstrate the importance of teaching young people to make healthy choices regarding their sexual behavior even before they become sexually active.

## Data Availability

Publicly available datasets were analyzed in this study. These data can be found here: https://www.uib.no/en/hbscdata/113290/open-access.
